# Development of a Multi-Stage Electroosmotic Flow Pump Using Liquid Metal Electrodes †

**DOI:** 10.3390/mi7090165

**Published:** 2016-09-14

**Authors:** Meng Gao, Lin Gui

**Affiliations:** Key Laboratory of Cryogenics, Technical Institute of Physics and Chemistry, Chinese Academy of Sciences, 29 Zhongguancun East Road, Haidian District, Beijing 100190, China; mgao@mail.ipc.ac.cn

**Keywords:** electroosmotic flow pump, liquid metal electrodes, microfluidics

## Abstract

Injection of liquid metal into a polydimethylsiloxane (PDMS) channel can provide a simple, cheap, and fast method to fabricate a noncontact electrode for micro electroosmotic flow (EOF) pumps. In this study, a multi-stage EOF pump using liquid metal noncontact electrodes was proposed and demonstrated for high-flow-velocity applications. To test the pumping performance of this EOF pump and measure the flow velocity, fluorescent particles were added into deionized (DI) water to trace the flow. According to the experimental results, the pump with a five-stage design can drive a water flow of 5.57 μm/s at 10 V, while the PDMS gap between the electrode and the pumping channel is 20 μm. To provide the guidance for the pump design, parametric studies were performed and fully discussed, such as the PDMS gap, pumping channel dimension, and stage number. This multi-stage EOF pump shows potential for many high-flow-velocity microfluidic applications.

## 1. Introduction

Electroosmotic flow (EOF) pumps have become attractive for a wide range of applications, including microfluidic actuation [1,2], biochemical analysis [3,4,5,6], and electronic cooling [7,8]. Most EOF pumps utilize contact electrodes to induce an electric field through the fluid channel to generate EOFs [9,10]. Due to the direct contact of these electrodes with the fluid in the channel, electrolysis can easily take place near the electrodes under direct current (DC) electric fields, leading to serious problems, such as bubble production, electrode erosion, and Joule heat formation [11,12,13]. If the contact electrodes are embedded inside the fluid channel, the bubbles will also block the microchannels, and decrease the flow rate greatly. Although an alternating current (AC) electric field has been demonstrated to suppress the electrolysis and bubble generation [4,8,14], it cannot offer continuous flows. In order to eliminate the electrolysis and offer continuous flows, noncontact electrodes have been developed in recent years.

The most well-known noncontact electrode is the gel-type salt bridge electrode [15,16], which is the simplest type of noncontact electrode reported, but is not suitable for long running times. The challenges of this noncontact electrode are to maintain compatibility between the gel and the metal electrodes and, at the same time, assure that the gel still holds the stability of mechanical property for long after curing. Recently, polyacrylamide-filled capillary [17,18], Nafion-coated Ag/Ag_2_O [19,20], alizarin paste-coated carbon paper [21,22], and polyaniline-wrapped aminated graphene [23] have been reported to fabricate noncontact electrodes for EOF pumps. These noncontact electrodes can enable stable and efficient pumping performance for a long time. However, the fabrication of these electrodes requires a relatively complex process, which is also expensive and time-consuming.

Due to the outstanding electrical and mechanical properties, liquid metal has been considered as a promising material to fabricate noncontact electrodes in microfluidics [24].The liquid metal is a kind of gallium-based alloy whose melting point is around room temperature. It can be easily and quickly injected into polydimethylsiloxane (PDMS) microchannels to form stable electrodes in any shape, at any location, because of its advantageous properties, including good flowability, deformability, low viscosity, and excellent wettability with PDMS [24,25,26]. In our previous work [27], we proposed a handy single-channel EOF pump using this liquid metal noncontact electrode. The minimum driven voltage of the EOF pump can be as low as 1.6 V with a 100-μm long pumping channel and 20-μm PDMS gap between the electrode and the pumping channel. There were no bubbles occurring on the electrode surfaces during pumping. However, the possible maximum flow velocity of this pump only reaches 270 µm/s before the 20-μm PDMS gap is damaged at 450 V (dielectric breakdown), which is not suitable for many high-flow-rate microfluidic applications. Moreover, the design of the liquid metal electrodes in this pump is also not convenient for the integration of the parallel pumping channels to improve the flow velocity.

**Figure 1:**
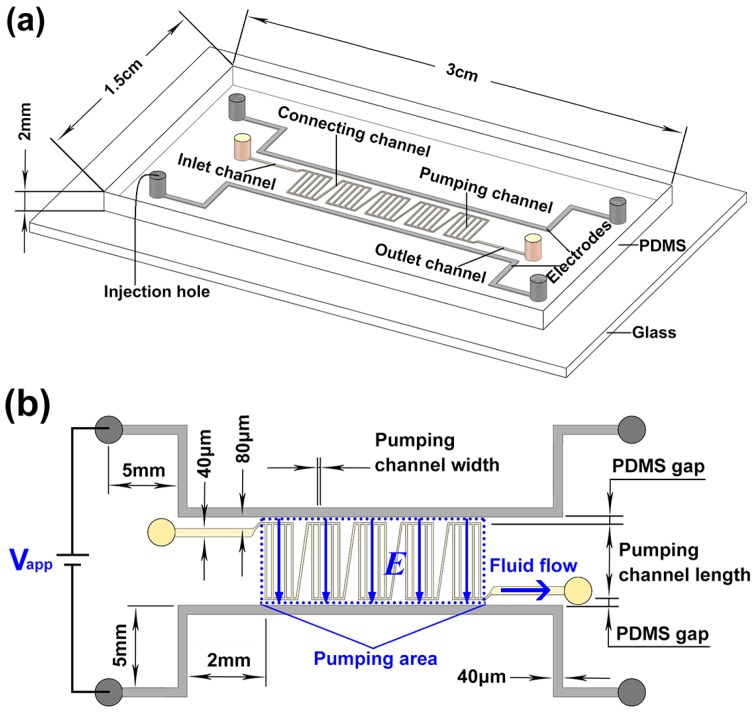
Schematic diagram of five-stage electroosmotic flow (EOF) pump. (**a**) Polydimethylsiloxane (PDMS)/glass microfluidic chip with ohm-shape liquid-metal electrodes. The PDMS slab is 3 cm long, 1.5 cm wide, and 2 mm thick. The chip substrate is a glass slide with 7.6 cm length, 2.5 cm width, and 1 mm thickness; (**b**) The working principle of the EOF pump. The electrode channel is 40 μm wide with both inlet and outlet 5 mm from the electrode head. The inlet and outlet fluid channels are 2 mm long and 40 μm wide, which are 80 μm far from the electrode head. All channels are 20 μm high. In this pump, the PDMS gap between adjacent two pumping channels has the same size as the width of the pumping channel.

In this work, a multi-stage EOF pump (shown in Figure 1) using the liquid metal noncontact electrode was developed for high-flow-rate microfluidic applications. To show the pumping performance of this pump, a deionized (DI) water suspension containing fluorescent particles was injected into the pumping channels to measure the pumping flow velocity. Parametric studies were performed in detail to investigate the effects of PDMS gap and pumping channel dimensions on the pumping performance.

## 2. Liquid Metal-Based Electroosmotic Flow (EOF) Pump

As shown in Figure 1a, PDMS is used to fabricate micro channels of the liquid metal-based EOF pump, and then bonded with a glass slide to form a microfluidic chip using plasma treatment. The chip is 3 cm long, 1.5 cm wide, and 2 mm thick. The two liquid metal microchannels are located in parallel near the pumping area with only a small PDMS gap separating the liquid metal microchannel and the ends of the parallel pumping channels. Five identical straight pumping channels are placed in parallel to form one stage, and five stages are connected in serial from the whole pumping area. The two electrode channels are designed vertically to the pumping channels to gain the maximum potential gradient along the pumping direction. For the convenience of liquid metal injection, both electrode channels are designed in an ohm shape. The channels between adjacent stages are not vertical to the electrode channels to reduce the reverse EOFs in it as much as possible. The inlet and outlet fluid channels are placed in parallel to the two electrode channels.

As shown in Figure 1b, when a voltage is applied, a parallel electric field will be induced in the pumping area (within the blue dashed box), generating EOFs along the pumping channels. In this EOF pump, the two electrode channels are extended by 2 mm (10 times of pumping channel length) from the edge of pumping area. An advantage of this extension design is that it can make the distribution of electric field inside the pumping area more uniform, which will help generate high-velocity EOFs in these pumping channels. To show the design guidance for the EOF pump, parametric studies were performed. The PDMS gap (20 μm, 30 μm, 40 μm, and 50 μm), pumping channel length (100 μm, 200 μm, and 500 μm) and width (20 μm, 30 μm, and 40 μm), pump stage number (single-stage, five-stage, and 10-stage) are fully discussed. The PDMS gap between adjacent pumping channels has the same size as the pumping channel width. The connecting channel has the same width, but is 1.4 times long as the pumping channel. The width and length of the inlet/outlet channel are 40 μm and 2 mm, respectively. The PDMS gap between the inlet/outlet channel and the electrode channel is 80 μm. The electrode channel is 40 μm wide. All channels are 20 μm high.

## 3. Experimental Details

### 3.1. Chip Fabrication

**Figure 2:**
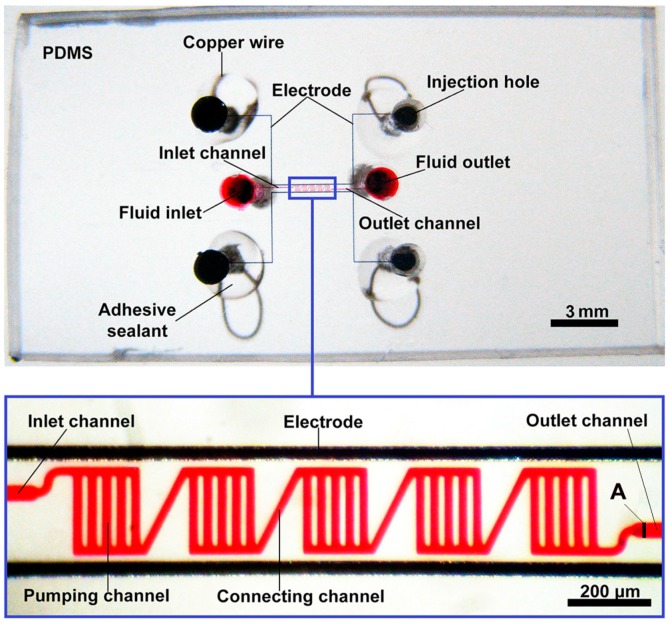
Photographs of the PDMS/glass chip for the five-stage EOF pump. The photo within the blue box shows the parallel pumping channels and the liquid metal electrodes of the pump. The pumping channels are filled with high red ink to mark their positions and shapes in the chip. In this pump, the PDMS gap is 20 μm. The pumping channel is 20 μm wide and 200 μm long. The connecting channel is 20 μm wide and 280 μm long. The inlet/outlet channel is 40 μm wide and 2 mm long. The electrode channel is 40 μm wide. All channels are 20 μm high. “A” represents the position of the pumping flow velocity measurement.

All chips were fabricated using soft-lithography [28]. Twenty micrometer thick SU-8 2025 (MicroChem, Westborough, MA, USA) layers were spin-coated on a 4-inch silicon wafer to fabricate the channel pattern masters. Sylgard 184 silicone elastomer (mixture of base and curing agent at a 10:1 ratio by weight, Dow Corning, Midland, MI, USA) was then prepared, poured onto the wafer, and degassed by vacuum. After cured, the PDMS was peeled off from the wafer with the channels patterned on one side of the PDMS slab. The patterned PDMS slab was irreversibly bonded with a glass slide using plasma treatment. Figure 2 shows the photographs of a PDMS/glass chip for the five-stage EOF pump, and the subfigure shows the details of the electrodes and pumping channels in the chip.

GaIn_20.5_Sn_13.5_ (weight percent, Ga 66%, In 20.5%, Sn 13.5%; melting point: 10.6 °C; Shanxi Zhaofeng Gallium Co., Ltd., Quanyang, China) was used to fabricate electrodes in this work. A syringe was used to inject GaIn_20.5_Sn_13.5_ into the electrode channels. Then, fine copper wires of 150 μm in diameter were inserted into the holes at the electrode channel ends, and kept good contact with the liquid metal inside. Finally, the wire joints were sealed to fasten the connection using an adhesive sealant (705 RTV Silicone Rubber, Kangda Chemical Co., Ltd., Liyang, China), as shown in Figure 2.

### 3.2. Measurement of Pumping Performance

To obtain a highly stable electroosmotic property, pumping channels were immediately filled with deionized (DI) water after chip bonding [29]. A high voltage sequencer (HVS448 6000D, LabSmith, Inc., Livermore, CA, USA) was used to offer high voltages (>60 V) and monitor the electric current at the same time. Low voltages (≤60 V) were offered by a DC power supply (U8032A, Keysight, Santa Rosa, CA, USA). 0.5-μm fluorescent polystyrene particles (Excitation wavelength (Ex) 542 nm, Emission wavelength (Em) 612 nm, 1% solids) from Duke Scientific Corporation (Palo Alto, CA, USA) were diluted 10,000 times in DI water, and then used as tracing particles for the flow speed detection. For the accurate measurement, the fluid inlet and outlet reservoirs of the chip were completely covered with a suitable water drop to balance the static pressure through the pumping channels after loading fluorescent particles. A fluorescence microscope (Axio Observer Z1, Carl Zeiss, Jena, Germany) was used to monitor the movements of fluorescent particles in the pumping channels during the experiments. Five particles with different distances to the outlet channel wall near the pumping area (position A shown in Figure 2) were randomly chosen and traced to calculate the mean flow velocity of the pumping area.

## 4. Results and Discussion

### 4.1. Pumping Velocity

**Figure 3:**
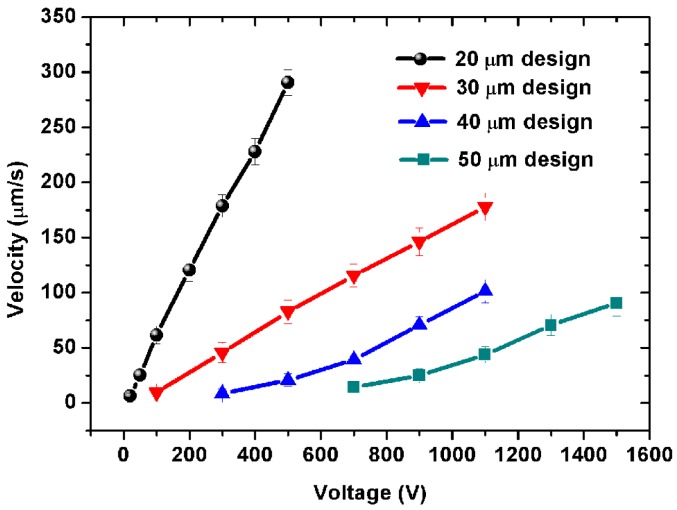
Experimental results of pumping velocity as a function of driving voltage with different pumping channel widths and PDMS gaps between the electrode and pumping channels in a five-stage pump. The PDMS gap between two adjacent pumping channels has the same size as the width of the pumping channel. For example, the 20-μm design has a 20-μm PDMS gap, 20-μm wide pumping channels, and 20-μm wide connecting channels. In these four pumps, the pumping channels and connecting channels are 200 μm and 280 μm long, respectively. The inlet and outlet channels are all 2 mm long, 40 μm wide. All channels are 20 μm high.

Figure 3 shows the pumping velocity in the outlet channel as a function of driving voltage with different PDMS gaps (or the pumping channel width, equal to the PDMS gap in the designs) in the five-stage pump. As shown in Figure 3, the PDMS gap is key to the pumping velocity. The pump with a smaller PDMS gap can drive the water flow at a lower voltage. The water flow of the 20 μm design pump can reach to 6.26 μm/s at 20 V, but the flow of the 50-μm design pump starts at 500 V. However, a smaller gap will increase the difficulty of fabrication greatly, and a larger gap will decrease the efficiency of the pump dramatically. In this work, the smallest PDMS gap is recommended to be 20 μm. Extreme tests show that a 20-μm PDMS gap will be damaged if the applied voltage reaches 2400 V. Before breaking down, the pump reaches its highest possible pumping flow velocity of 1.2 mm/s.

**Figure 4:**
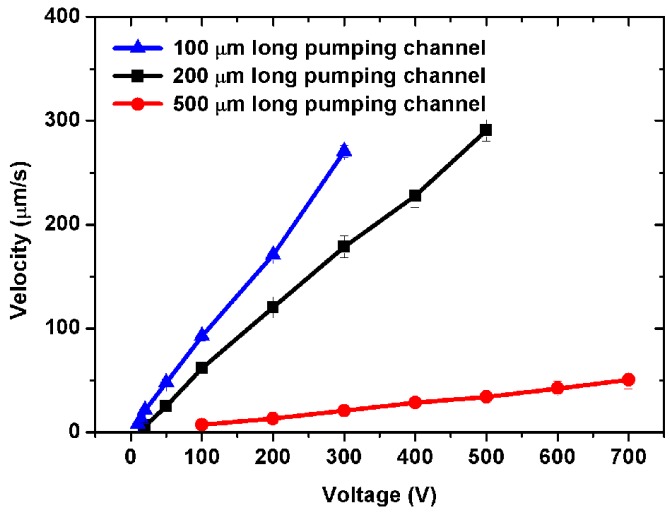
Experimental results of pumping velocity as a function of driving voltage with different pumping channel length in a five-stage pump. The PDMS gap is 20 μm. The pumping and connecting channels are 20 μm wide. The connecting channels are 1.4 times as long as the pumping channels. The inlet and outlet channels are 2 mm long, 40 μm wide. All channels are 20 μm high.

Figure 4 shows the pumping velocity in the outlet channel as a function of the driving voltage with different pumping channel length in the five-stage pump. 100-μm, 200-μm and 500-μm long pumping channels are considered. The PDMS gap is 20 μm. The pumping channels are 20 μm wide. As shown in Figure 4, the pumping velocity increases with the increment of the driving voltage linearly. The 100-μm design can drive a flow up to 5.57 μm/s at 10 V, and the 200-μm design can drive a flow up to 6.26 μm/s at 20 V. However, the 500-μm design can only begin to drive the flow when the voltage reaches as high as 100 V. Thus, the pumping channel length plays an important role on the pumping velocity. Smaller pumping channel length will help the reduction of the driving voltage. But it will also shrink the pumping area (shown in Figure 1) and decrease the pumping energy. Thus, 100 μm is now recommended to be the low limit of the pumping length of the 20-μm PDMS gap design. Extreme tests show that the 20-μm PDMS gap will be damaged if the applied voltage reaches 2300 V (100-μm long pumping channels). Before breaking down, the pump reaches its highest possible pumping flow velocity of 1.4 mm/s.

As compared with the pump in our previous work (minimum voltage 1.6 V, maximum velocity 270 µm/s at 450 V in 100 μm long pumping channel [27]), although this pump did not lower the minimum driving voltage (≥10 V). The reason is that the liquid metal electrodes in our previous work [27] can induce much higher parallel electric fields in the pumping channel than the electrodes in this work at the same voltage (Supplementary Material). However, the pump in this work has greatly increased the maximum pumping capacity at high voltages. It reaches its highest possible velocity at 1.4 mm/s when the applied voltage reaches its limit 2300 V. While the previous pump can only reach its maximum 270 µm/s when the applied voltage reaches its limit 450 V. Thus, the pump designed in this work can be as a good choice for some high-flow-rate on-chip applications. Moreover, the liquid metal electrodes of the multi-stage pump are much more convenient for changing the number of pumping channels/stages to adjust the pumping capacity than those of just the single-channel pump in [27].

**Figure 5:**
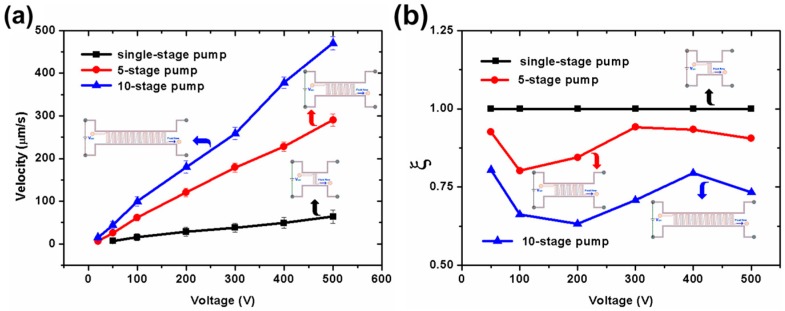
Experimental results of pumping velocity vs. pump stage number. (**a**) Pumping velocity as a function of driving voltage with different pump stage number; (**b**) Pumping efficiency ξ as a function of driving voltage with different pump stage number. The PDMS gap is 20 μm. The pumping channels are 200 μm long, 20 μm wide. The connecting channels are 280 μm long, 20 μm wide. The inlet and outlet channels are 2 mm long, 40 μm wide. All channels are 20 μm high.

Figure 5 shows the pumping velocity in the outlet channel as a function of driving voltage with different pump stage numbers. Single-stage, five-stage, and 10-stage designs are performed in this work. The PDMS gap is 20 μm. The pumping channels are 20 μm wide and 200 μm long. It can be seen clearly from Figure 5a that the pumping velocity will increase with the increment of the stage number of the pump. When a voltage of 20 V is applied, the five-stage design and the 10-stage design can start to drive water flows of 6.26 and 7.24 μm/s, respectively. However, the single-stage design only begins to drive the flow when the voltage reaches as high as 50 V. When a high voltage of 500 V is applied, the five-stage design and the 10-stage design can, respectively, drive 3.52 times and 6.33 times higher pumping flow velocity than the single-stage design. In other words, adding the stage number of the pump can help to effectively improve the pump energy, generating higher pumping flow velocity.

Since higher pump stage numbers will also cause higher fluid flow resistance, simply increasing the stage number is not always the best way to increase the pumping efficiency. To evaluate the comprehensive pumping performance of the EOF pump, a parameter ξ describing the pumping efficiency was given in this part. The efficiency ξ is defined as the ratio of the pumping velocity between the n-stage pump (μ*_n_*) and the single-stage pump (μ_1_) at a certain applied voltage (ξ = μ*_n_*/(*n*·μ_1_), *n* ≥ 1). Figure 5b shows the pumping efficiency of the pump as a function of driving voltage with different pump stage numbers. As shown in Figure 5b, the five-stage pump has decreases of 5.88%–19.78% in pumping efficiency as compared with the single-stage pump from 50 to 500 V. However, the 10-stage pump has large decreases of 19.59%–36.76% in pumping efficiency as compared with the single-stage pump.

### 4.2. Electric Current and Power Consumption

**Figure 6:**
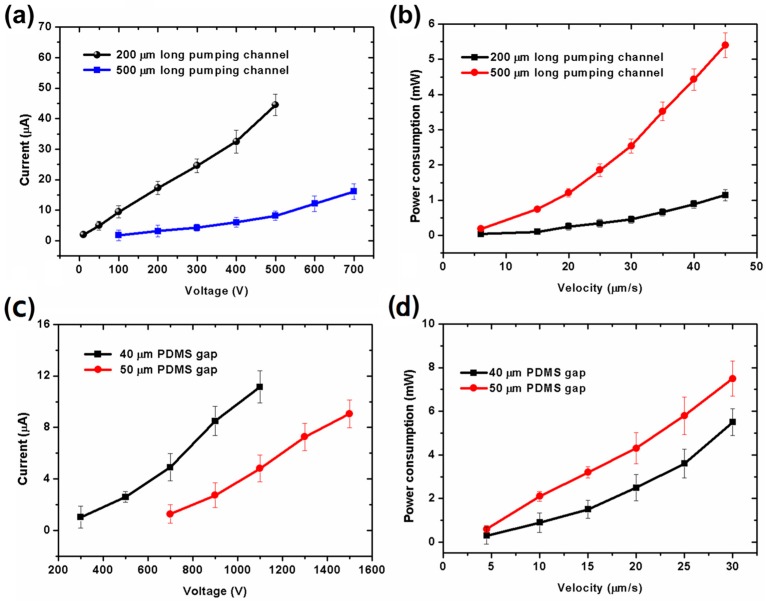
Experimental results of electric current through PDMS gap in a five-stage pump. (**a**) Current as a function of applied voltage with different pumping channel length; (**b**) Power consumption as a function of velocity with different pumping channel lengths. The pumping and connecting channels are all 20 μm wide. The connecting channels are 1.4 times as long as the pumping channels. The PDMS gap between electrode and pumping channels is 20 μm. The PDMS gap between adjacent two pumping channels is 20 μm. The inlet and outlet channels are 2 mm long, 40 μm wide. All channels are 20-μm high; (**c**) Current as a function of applied voltage with different PDMS gaps between the electrode and pumping channels; (**d**) Power consumption as a function of velocity with different PDMS gaps between electrode and pumping channels. The length and width of pumping channels are 200 μm, 40 μm, respectively. The connecting channels are 280 μm long, 40 μm wide. The inlet and outlet channels are 2 mm long, 40 μm wide. All channels are 20 μm high. The PDMS gap between two adjacent pumping channels is equal to the width of the pumping channel.

Electric current is a dominant factor for the Joule heating generated in the EOF pumps. Figure 6a,c show the electric current as a function of applied voltage with pumping channel length and different PDMS gap in the five-stage pump during pumping. As shown in Figure 6a,c, there are several μA to several tens of μA of electric current through the PDMS gap, which almost cannot increase the temperature of fluid in the pumping channels. As shown in Figure 6b,d, the power consumption of the pump is just several mW, which can drive water flows with several μm/s to tens of μm/s. It can also be seen from Figure 6 that the electric current increases with the applied voltage. In other words, when a certain voltage is applied, the pump with a smaller PDMS gap or shorter pumping channel will have a higher current through the PDMS gap. That means there will be more free charges in the electrical double layer to drag fluids in the pumping channels, and pass through the PDMS gap under the applied electric field. Then, the pump will get higher-velocity electroosmotic flows in the pumping channels (shown in Figure 3 and Figure 4).

## 5. Conclusions

A liquid-metal-filled microfluidic channel as a noncontact electrode has found attractive utility in EOF pumps due to its ease of fabrication and integration. In this study, a multi-stage EOF pump using this liquid metal electrode has been proposed and demonstrated. The liquid metal electrode was designed in an ohm shape, and vertically located to each end of the multi-stage parallel pumping channels, having no direct contact with these pumping channels. To test the pumping performance, the pumping flow velocity was experimentally measured using the fluorescence tracing technique in this research. The PDMS gap, pumping channel dimension, and pump stage number were considered to perform parametric studies. According to the results, the liquid-metal-filled microchannel, combined with the PDMS gap as a noncontact electrode, can help the EOF pump to prevent water electrolysis and reduce Joule heating. In the near future, the pump should have potential uses in many high-flow-rate microfluidic applications. Moreover, the liquid-metal noncontact electrodes should be promising microdevices in other microfluidic applications, such as dielectrophoresis, electrowetting, and other electro-actuated microsystems.

## Supplementary Materials

The following are available online at www.mdpi.com/2072-666X/7/9/165/s1, Figure S1: Numerical results of electric field (horizontal plane at middle of pumping channel) of the single-channel pump [27] and the multi-stage channel pump from numerical model; Figure S2: Mean electric field strength in the pumping channel of the single-channel pump [27] and the multi-stage channel pump as a function of applied voltages; Video S1: Fluorescent particle movements in the pumping channels of 20 μm design pump with 5-stage pumping channels at 50 V; Video S2: Fluorescent particle movements in the pumping channels of 20 μm design pump with 10-stage pumping channels at 100 V.
