# Supplementary material for: Development of a Multi-Stage Electroosmotic Flow Pump Using Liquid Metal Electrodes
Source: Micromachines (Basel). 2016 Sep 14;7(9):165. doi: 10.3390/mi7090165 (PMC6190331; doi:10.3390/mi7090165)
Supplement: Supplementary file 1 [file micromachines-07-00165-s001.pdf]

Supplementary Materials: Development of a Multi-Stage Electroosmotic Flow Pump Using Liquid Metal Electrodes

Meng Gao and Lin Gui

1. Numerical Simulation

For better understanding how the multi-stage electroosmotic flow (EOF) pump works, numerical simulation of the electric field distribution in the pumping channels was performed in this section. The simulation aims to compare the electric field generated by the liquid metal electrodes between the single-channel pump [1] and the multi-stage channel pump in this work. The simulation work is helpful for us to understand the performance of the both pumps. The commercial software COMSOL Multiphysics 3.5 (COMSOL Inc., Stockholm, Sweden) was used to perform the simulation in this research.

2. Numerical Model

In a single-channel EOF pump, the electroosmotic flow velocity at the solid-liquid interface of the pumping channel can be written as:

$$U = \mu_{eo}E \qquad \text{(S1)}$$

where E is the electric field strength along the pumping channel wall, and μ_{eo} is the fluid electroosmotic mobility. The electroosmotic mobility (μ_{eo}) is a physical property parameter, which is only dependent on the types of fluid and channel wall, temperature, pH, and ion concentration in the fluid [2]. When deionized (DI) water is used as the working fluid in the polydimethylsiloxane (PDMS) pumping channel at room temperature, the electroosmotic mobility (μ_{eo}) is constant. Then, the electroosmotic flow velocity can be determined by the electric field strength (E) along the pumping channel wall. Thus, the electric field strength (E) is the only factor for the pumping performance of this EOF pump.

With multi-stage parallel pumping channels, the EOF pump can drive electroosmotic flow with velocity as follows:

$$U_n = n\mu_{eo}E \qquad \text{(S2)}$$

where n is the number of the parallel pumping channels. The fluid flow resistance and pressure driven flow in the pumping channels are not considered in Equations (S1) and (S2).

3. Numerical Results

Figure S1 shows the numerical results of the electric field (horizontal plane at the middle of the pumping channel) of the single-channel pump [1] and the multi-stage channel pump from the numerical model. All pumping channels in this simulation are 20 μm wide, 20 μm high, and 100 μm long. The PDMS gap between the electrodes and the pumping channels is 20 μm. The multi-stage channel pump has five parallel pumping channels with 20 μm PDMS gap between them. The electrode channels are all 20 μm wide and 20 μm high, and extended by 1 mm (10 times of pumping channel length) from the edge of pumping area. The chip is 2 cm wide, 2 cm long, and 2 mm thick. A voltage of 10 V is applied at the liquid metal electrodes.

As shown in Figure S1, the liquid metal electrodes can generate an electric field parallel to the pumping channel in these two pumps. It can be seen clearly from Figure S1 that the single-channel pump has much higher strength of the electric field than the multi-stage pump. In other words, the liquid metal electrodes of the single-channel pump are more efficient to induce a high electric field than that of the multi-stage pump. As shown in Figure S2, the electric field strength of the single-channel pump is 2.4–2.6 times of that of the multi-stage pump when the voltage is ranged

from 10–90 V. Thus, to obtain a higher flow velocity than the single-channel pump at the same voltage, the multi-stage pump should have ≥3 parallel pumping channels, as shown in Equation (S2). Moreover, the liquid metal electrodes of the multi-stage pump are much more convenient for changing the number of parallel pumping channels to change the pumping capacity than those of the single-channel pump. When required to offer relatively high-flow velocity fluid for many microfluidic applications, the multi-stage pump can be a good substitute for the single-channel pump.

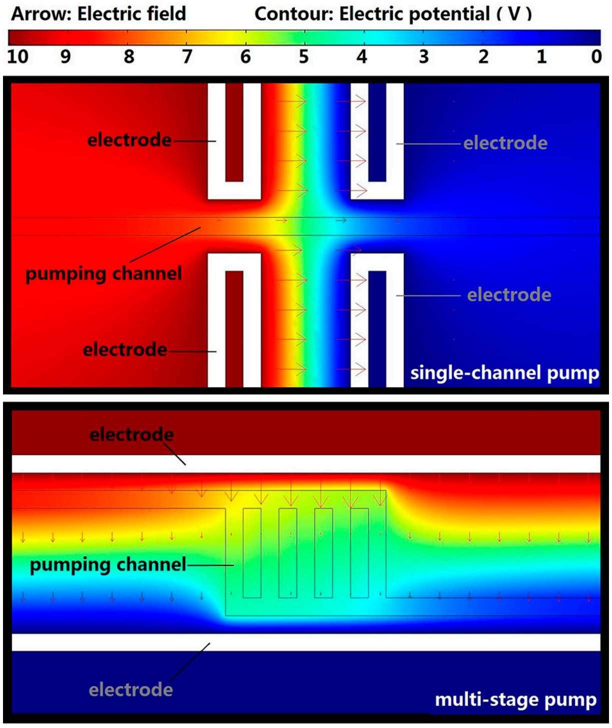

Figure S1. Numerical results of electric field (horizontal plane at middle of pumping channel) of the single-channel pump [1] and the multi-stage channel pump from numerical model.

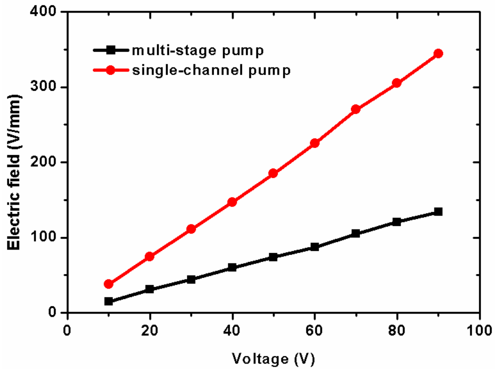

Figure S2. Mean electric field strength in the pumping channel of the single-channel pump [1] and the multi-stage channel pump as a function of applied voltages.

References

1. Gao, M.; Gui, L. A handy liquid metal based electroosmotic flow pump. *Lab Chip* **2014**, 14, 1866–1872.
2. Li, D. *Electrokinetics in Microfluidics*; Elesevier: Amsterdam, Netherlands, 2004.
